# Compound heterozygous mutations in glycyl-tRNA synthetase are a proposed cause of systemic mitochondrial disease

**DOI:** 10.1186/1471-2350-15-36

**Published:** 2014-03-26

**Authors:** Hugh J McMillan, Jeremy Schwartzentruber, Amanda Smith, Suzie Lee, Pranesh Chakraborty, Dennis E Bulman, Chandree L Beaulieu, Jacek Majewski, Kym M Boycott, Michael T Geraghty

**Affiliations:** 1 Children’s Hospital of Eastern Ontario Research Institute, University of Ottawa, 401 Smyth Rd, Ottawa, ON K1H 8 L1, Canada; 2 McGill University and Genome Quebec Innovation Centre, Montréal, QC, Canada; 3 Ottawa Hospital Research Institute, University of Ottawa, Ottawa, ON, Canada; 4 Department of Human Genetics, McGill University, Montréal, QC, Canada

**Keywords:** Glycyl-tRNA synthetase, Amino acyl-tRNA synthetase, Cardiomyopathy, Charcot-Marie-tooth disease

## Abstract

**Background:**

Glycyl-tRNA synthetase (GARS) is an aminoacyl-tRNA synthetase (ARS) that links the amino acid glycine to its corresponding tRNA prior to protein translation and is one of three bifunctional ARS that are active within both the cytoplasm and mitochondria. Dominant mutations in *GARS* cause rare forms of Charcot-Marie-Tooth disease and distal spinal muscular atrophy.

**Case presentation:**

We report a 12-year old girl who presented with clinical and biochemical features of a systemic mitochondrial disease including exercise-induced myalgia, non-compaction cardiomyopathy, persistent elevation of blood lactate and alanine and MRI evidence of mild periventricular leukomalacia. Using exome sequencing she was found to harbor compound heterozygous mutations within the glycyl-tRNA synthetase (*GARS*) gene; c.1904C > T; p.Ser635Leu and c.1787G > A; p.Arg596Gln. Each mutation occurred at a highly conserved site within the anticodon binding domain.

**Conclusion:**

Our findings suggest that recessive mutations in *GARS* may cause systemic mitochondrial disease. This phenotype is distinct from patients with previously reported dominant mutations in this gene, thereby expanding the spectrum of disease associated with GARS dysregulation.

## Background

Aminoacyl-tRNA synthetases (ARS) are nuclear-encoded, ubiquitously expressed enzymes that are essential for cytosolic and mitochondrial protein translation [1]. Each ARS links a specific amino acid to its corresponding tRNA, a process known as aminoacylation or “charging” of tRNA. Aminoacylation must take place before protein translation can occur. ARS are being increasingly recognized as having important secondary functions that include regulation of transcription, translation, splicing and apoptosis [2]. ARS mutations have been implicated in a wide range of human diseases. There are 37 different ARS that are divided into three groups depending upon the site of tRNA aminoacylation. Cytoplasm-specific ARS are active within the cell nucleus and cytoplasm. The tRNA-amino acid complexes are transported within the cytoplasm to ribosomes where they facilitate protein translation. Mitochondria-specific ARS are a distinct group of nuclear-encoded proteins that are imported into the mitochondria to carry out tRNA aminoacylation at that site. Three bifunctional ARS have been described that are active in both the cytoplasm and mitochondria.

Glycyl-tRNA synthetase (GARS) is a bifunctional ARS. Structurally, the functional enzyme exists as a homodimer with three functional domains [3]. *GARS* mutations have been linked to autosomal-dominant Charcot-Marie-Tooth disease type 2D (CMT2D) and distal spinal muscular atrophy type 5 (dSMA5) [3,4]. Numerous mechanisms have been postulated as to how dysregulation of GARS may give rise to an axonal neuropathy including loss of function, protein aggregation, loss of a secondary ‘housekeeping’ function, and/or mitochondrial dysfunction [5]. The latter is particularly appealing in light of the activity of GARS within mitochondria [1] as well as the knowledge that other inherited neuropathies have been linked to mitochondrial dysfunction: *MFN2* mutations causing CMT2A [6] and *GDAP1* mutations causing CMT4A or CMT2K [7].

We report a girl who presented with clinical and biochemical features of a systemic mitochondrial disease and using exome sequencing we identified compound heterozygous mutations within the *GARS* gene. We propose that her phenotype is secondary to recessive mutations in *GARS*, thereby expanding the phenotypic spectrum associated with mutation of this gene.

## Case presentation

### Patient description

A 12 year old girl initially presented at 6 years of age with exercise intolerance. She was born to non-consanguineous healthy parents. She has two younger, healthy brothers although one has autistic spectrum disorder. She presented with shortness of breath with low-intensity aerobic exercise such as jogging or biking. She had no chest pain or palpitations. Cardiac examination at 7 years old noted a normal clinical examination however her electrocardiogram revealed biventricular hypertrophy. Subsequent echocardiogram and cardiac MRI identified thickening of the posterior left ventricle (LV) wall, apex and septum consistent with a non-compaction cardiomyopathy. Bilateral ventricular systolic and diastolic function was normal with a LV ejection fraction of 63%. Stress testing confirmed a normal baseline heart rate, blood pressure and a normal response to exercise. Pulmonary function tests were normal. Biochemical testing revealed normal serum creatine kinase (CK) with a slight elevation of serum troponin T. Genetic testing included normal *SCN5A* sequencing, chromosomal microarray and hypertrophic and dilated cardiomyopathy panel (GeneDx, Gaithersburg, MD).

Over the next year, she reported exercise-induced myalgia. She had no muscle weakness, cramping or pigmenturia. She could perform short bursts of anaerobic activity without difficulty however, sustained activity would elicit muscle pain. Biochemical testing was abnormal on multiple occasions including: plasma lactate (2.3 – 4.6 mmol/L; normal 0.5 - 2.2 mmol/L) and plasma alanine (603 - 841 μmol /L; normal 152 - 547 μmol/L). Her acylcarnitine profile and carnitine levels (free and total) were normal as was urine organic acid analysis. Repeated serum CK, liver and renal function were normal. Neuromuscular assessment at 9 years of age showed her cranial nerves, muscle power, reflexes, sensory testing and coordination to be within normal limits. Gower manoeuvre was negative and gait was normal. Electrodiagnostic testing confirmed normal right median and sural nerve sensory responses and normal right median and tibial nerve motor responses. Concentric needle electromyography of her right quadriceps was normal.

Muscle biopsy of the quadriceps performed at 9 years of age revealed a preponderance of type 1 fibers although no other microscopic, histochemical or ultrastructural abnormalities were apparent. Muscle respiratory chain enzyme testing and muscle mitochondrial DNA sequencing was normal. MRI of the brain at 10 years of age revealed abnormal T2 and T2FLAIR hyperintensity in the periventricular and trigonal white matter bilaterally. MR spectroscopy of the basal ganglia and subcortical white matter was normal. MRI of the proximal leg muscles was unremarkable. Treatment with ubiquinone, vitamin B50 complex, levocarnitine and ubiquinol were started at 10 years old without apparent clinical effect. Creatine monohydrate was added several months later after which she reported a sustained, subjective clinical improvement in her exercise tolerance.

Neuromuscular evaluation at 12 years old was significant for bilateral extensor hallucis longus and extensor digitorum brevis weakness (4/5). Strength testing of all other muscles was within normal limits. Cranial nerve testing, deep tendon reflexes, sensory testing and coordination were within normal limits. Repeat neurophysiological testing revealed a reduction in her right common peroneal nerve motor response due to a slight CMAP amplitude reduction of 2.1 mV (normal >2.4 mV). Motor responses at the left peroneal, right tibial, right median and ulnar nerves were normal. Sensory responses at the right median, ulnar, superficial peroneal and sural nerves were normal. Concentric needle EMG of the right tibialis anterior and medial gastrocnemius was normal. Her most recent cardiac evaluation at 12 years old identified two new findings: electrocardiogram identified a new subclinical Wolf-Parkinson-White pre-excitation that was not noted on prior studies as well as evidence for LV diastolic dysfunction on echocardiogram.

Her medical history was otherwise unremarkable. She was born at term with no complications. Early milestones were appropriate; she sat at 6 months old, pulled to stand by 12 months and walked independently by 18 months of age. Her growth parameters were stable; height (just <50^th^ percentile) and weight (just < 25^th^ percentile) following along her percentile curves from infancy. She has never had any seizures, headaches or endocrine dysfunction. She has no oculobulbar symptoms and no sensory or autonomic dysfunction. Her visual acuity and hearing were normal. She excelled academically, achieving high grades in a gifted program.

### Exome sequencing and analysis

We followed standard manufacturer protocols to perform target capture with the Agilent SureSelect All Exon 50 MB (V3) exome enrichment kit and sequencing of 100 bp paired end reads on Illumina Hiseq 2000, which generated over 12.4 Gb of data for the proband. We removed adaptor sequences and quality trimmed reads using the Fastx toolkit (http://hannonlab.cshl.edu/fastx_toolkit/) and then used a custom script to ensure that only read pairs with both mates present were subsequently used. Reads were aligned to hg19 with BWA 0.5.9 [8] and indel realignment was done using the GATK [9]. Duplicate reads were then marked using Picard (http://picard.sourceforge.net/) and excluded from downstream analyses. We assessed coverage of consensus coding sequence (CCDS) bases using the GATK, which showed that all samples had >91% of CCDS bases covered by at least 10 reads, and >85% of CCDS bases covered by at least 20 reads. Single nucleotide variants (SNVs) and short insertions and deletions (indels) were called using samtools mpileup [10] with the extended base alignment quality (BAQ) adjustment (-E), and were then quality filtered to require at least 20% of reads supporting the variant call. Variants were annotated using both Annovar [11] and custom scripts to identify whether they affected protein coding sequence, and whether they had previously been seen in dbSNP132, the 1000 genomes dataset (Nov 2011), the NHLBI GO exomes, or in approximately 500 exomes previously sequenced at our center.

**Figure 1:**
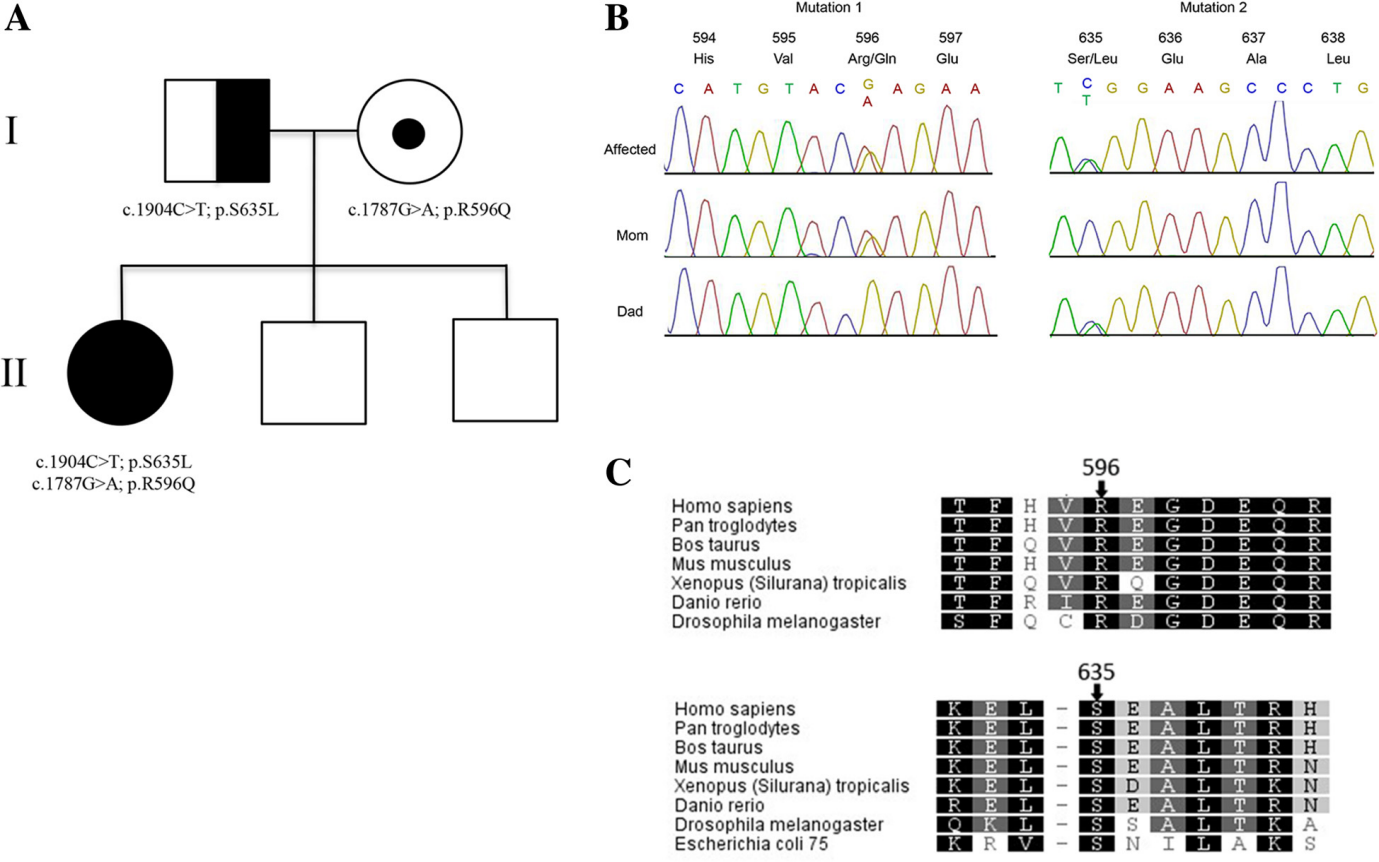
**Sanger sequencing and segregation. (A)** Pedigree of family with *GARS* mutations showing segregation of the two mutations found: c.1904C > T; p.Ser635Leu and c.1787G > A; p.Arg596Gln (NM_002047.2). The proband (solid circle) is a compound heterozygous with clinical features of a systemic mitochondrial disease. Her father (half shaded) shows mild sensorimotor polyneuropathy with axonal features. Her mother (small circle) is a carrier. **(B)** Sanger sequencing validation of *GARS* mutations identified by exome sequencing. **(C)** Conservation of the Arginine residue at position 596, and Serine residue at position 635 in the GARS protein.

Given the suspicion of a mitochondrial disorder based on the patient’s clinical phenotype, we first filtered the list of non-synonymous variants to retain only those present in genes in the MitoCarta Inventory of Mammalian Mitochondrial Genes and which were seen in 7 or fewer internal control exomes (of ~500) and at less than 1% frequency in the 1000 genomes and NHLBI GO exome databases. There were 13 genes with single heterozygous variants, and a single gene, *GARS*, with two rare heterozygous variants. The two *GARS* variants, NM_002047.2: c.1904C > T (p.Ser635Leu) and c.1787G > A (p.Arg596Gln) occur at highly conserved positions (Figure 1) and are predicted to be deleterious by both SIFT [12] (scores 0.01 and 0.00, respectively) and PolyPhen2 [13] (scores 0.94 and 1.00, respectively). We then analyzed the remaining exome data and no convincing disease-causing variants were identified in any other genes relevant to previously reported neuromuscular disorders.

We identified our patient to have a sequence variant in the *MIB1* (mindbomb E3 ubiquitin protein ligase 1) gene which has been linked to non-compaction cardiomyopathy. Our patient’s *MIB1* sequence variant has not been previously reported but did occur in a highly conserved region of this gene. We confirmed that the proband’s father carries the same *MIB1* sequence variant although he shows no evidence of cardiomyopathy on clinical examination or echocardiogram.

### Variant validation

Sanger sequencing was used to validate the variants in *GARS* and to evaluate segregation in the family. Blood samples were obtained and DNA was extracted from the patient as well as her parents and two unaffected siblings. PCR amplification and sequencing was performed with primers 5′CAGATGATCCACCTACCTCAG3′ and 5′ATAACACAGGAAACTGGTTTGTC-3′ to test the c.1787G > A variant. PCR amplification was performed using 5′AGTGAAGATTTGGATTCCCG-3′ and 5′GGACTTGAGAATCTGGGCTC3′ primers and Sanger sequencing was done using 5′AAGAAGCAGTACACATTTCTAAG-3 and 5′GTAAGACAGTAGTTAGATAAC-3 primers, to test the c.1904C > T variant.

Sanger sequencing confirmed the presence of these variants in the proband. The parents were each heterozygous for one of the mutations; c.1787G > A was inherited from her mother and c.1904C > T was inherited from her father (Figure 1). The c.1904C > T mutation has previously been reported to be disease-causing [4,14], the reported patient exhibited a clinical phenotype characterized by adolescent-onset foot deformity necessitating an orthopedic referral at the age 27 years old.

### Examination of family members

Given the findings of recessive mutations in *GARS* and the knowledge that heterozygous mutations in this gene can cause disease, the family was evaluated in detail. The proband’s father reported no weakness or sensory deficits when examined at 55 years old. His clinical examination was entirely within normal limits. His electrodiagnostic testing, however, revealed evidence of a mild sensorimotor polyneuropathy with axonal features. Bilateral sural and superficial peroneal nerve sensory amplitudes were low. His common peroneal and tibial motor amplitudes were within normal limits. Concentric needle EMG of his right extensor digitorum brevis and abductor hallucis revealed fibrillation potentials and positive sharp waves in addition to chronic neurogenic changes. Needle EMG of his right tibialis anterior also revealed chronic neurogenic changes. The proband’s mother reported no functional difficulty. Her clinical examination, echocardiogram and nerve conduction studies at age 47 years old were within normal limits. Needle EMG was not performed. The proband’s two younger brothers had no significant findings on clinical examination or nerve conduction testing.

## Discussion

Aminoacyl tRNA synthetase mutations are emerging as an important cause of rare childhood and adult diseases. Autosomal dominant mutations within several ARS are known to cause distal motor neuropathy or polyneuropathies, including cytoplasmic ARS such as alanyl-tRNA synthetase (AARS) [15], tyrosyl-tRNA (YARS) [16] and lysyl-tRNA synthetase (KARS) [17] as well as the bifunctional GARS [3]. Additionally, an increasing number of autosomal recessive ARS mutations have been linked to severe clinical phenotypes affecting the central nervous system and other highly metabolically active tissues. Mutations within the mitochondrial aspartyl-tRNAsynthetase (DARS2) have been linked to a syndrome of progressive spastic ataxia with MRI evidence of diffuse subcortical leukoencephalopathy with brainstem and spinal cord involvement [18,19]. Mutations within another mitochondrial ARS, tyrosyl-tRNA synthetase (YARS2) have been identified in a patient presenting with lactic acidosis, sideroblastic anemia and myopathy associated with a severe deficiency in respiratory chain enzyme function [20]. Our patient demonstrated similar, albeit milder, features of periventricular leukoencephalopathy, lactic acidosis and myalgia suggesting evidence of mitochondrial dysfunction. Each of the two *GARS* mutations occurred at a highly conserved site within the anticodon binding domain; mutations within the anticodon domain have been previously linked to more severe, earlier onset forms of disease [4].

Dominant mutations in *GARS* are a recognized cause of both CMT2D and dSMA5 and our findings suggest that recessive mutations in this gene are associated with mitochondrial disease. Mouse modeling supports our clinical observation that there are fundamental differences in disease phenotype and mechanism in autosomal dominant versus autosomal recessive *GARS*-associated disease [21]. While mice that were heterozygous for a missense mutation in *GARS* show a dominant-negative or toxic gain-of-function effect, mice that are homozygous for *GARS* mutations appear to show some additional loss-of-function effect that is not observed in the heterozygous state [21]. Finally, we cannot exclude the possibility that elements of our proband’s phenotype could secondary to more than GARS dysregulation and that a second concomitant disease is also present. The sequence variant in the *MIB1* gene for example raises suspicion that the non-compaction cardiomyopathy could be related to dysfunction in this gene and may or may not be related to the GARS gene dysfunction. However, even if the cardiomyopathy is due to this second gene, there is still substantial evidence for mitochondrial dysfunction in this girl given her persistent lactic acidosis, elevated serum alanine, exercise-induced myalgia and white matter changes on MRI brain.

## Conclusions

Our findings suggest that recessive mutations in *GARS* cause systemic mitochondrial disease. Given our patient’s clinical phenotype, the location of each of the two mutations in *GARS* and data from the mouse models, we postulate that the clinical phenotype of the patient reported may be explained by a loss-of-function mechanism due to impaired glycyl-tRNA aminoacylation. This phenotype reported here and associated with recessive mutations is distinct from patients with previously reported dominant mutations in this gene, thereby expanding the spectrum of disease associated with GARS dysregulation.

## Consent

Members of the study family consisted of the proband, mother, father, and siblings. Parents provided informed consent for themselves and their children to be enrolled in the Finding of Rare Disease Genes (FORGE) Canada study. The Research Ethics Board of the Children’s Hospital of Eastern Ontario approved this study in accordance with the Declaration of Helsinki. A copy of the written consent is available for review by the Editor of this journal.

## Abbreviations

ARS: Aminoacyl-tRNA synthetases; GARS: Glycyl-tRNA synthetase; SIFT: Sorting Intolerant From Tolerant; PolyPhen-2: Polymorphism Phenotyping; EMG: Electromyography; CMAP: Compound muscle action potential.

## Competing interests

The authors declare that they have no competing interests.

## Author’s contributions

HJM and MTG wrote the manuscript. MTG, PC and KB designed and coordinated the study. HJM, SL, and MTG provided subspecialist consultation services, serial clinical examinations and diagnostic testing. AS and DEB completed Sanger sequencing, modeling and conservation of GARS mutations. JS and JM carried out analysis of the next-generation sequencing data. All authors read and approved the final manuscript.

## Pre-publication history

The pre-publication history for this paper can be accessed here:

http://www.biomedcentral.com/1471-2350/15/36/prepub
